# Monocyte Signal Transduction Receptors in Active and Latent Tuberculosis

**DOI:** 10.1155/2013/851452

**Published:** 2013-01-20

**Authors:** Magdalena Druszczynska, Marcin Wlodarczyk, Beata Janiszewska-Drobinska, Grzegorz Kielnierowski, Joanna Zawadzka, Magdalena Kowalewicz-Kulbat, Marek Fol, Piotr Szpakowski, Karolina Rudnicka, Magdalena Chmiela, Wieslawa Rudnicka

**Affiliations:** ^1^Department of Immunology and Infectious Biology, Institute of Microbiology, Biotechnology and Immunology, Faculty of Biology and Environmental Protection, University of Lodz, Banacha 12/16, 90-237 Lodz, Poland; ^2^Regional Specialized Hospital of Tuberculosis, Lung Diseases and Rehabilitation, Szpitalna 5, 95-080 Tuszyn, Poland

## Abstract

The mechanisms that promote either resistance or susceptibility to TB disease remain insufficiently understood. Our aim was to compare the expression of cell signaling transduction receptors, CD14, TLR2, CD206, and **β**2 integrin LFA-1 on monocytes from patients with active TB or nonmycobacterial lung disease and healthy individuals with *M.tb* latency and uninfected controls to explain the background of the differences between clinical and subclinical forms of *M.tb* infection. A simultaneous increase in the expression of the membrane bound mCD14 receptor and LFA-1 integrin in patients with active TB may be considered a prodrome of breaking immune control by *M.tb* bacilli in subjects with the latent TB and absence of clinical symptoms.

## 1. Introduction

There is an urgent need to identify the factors that secure protective immunity to *Mycobacterium tuberculosis* (*M.tb*) for the humans exposed to this pathogen including those who have been immunized with the attenuated *M. bovis *BCG vaccine. An outstanding variation in the profile of *M.tb* infections has been documented [1]. *M.tb* is a pathogen characterized by the ability to persist within humans for a long period of time. Yet, more than 90% of the *M.tb* infected people never develop active TB, although they remain latently infected lifelong. The immune response to *M.tb* during latency is poorly explored. The development of clinical symptoms of TB takes place years or even decades after the exposure to *M.tb* as a consequence of failed immunity or/and pathogenic host inflammatory response to virulent mycobacteria [2]. There is also a small group of people, naturally resistant to TB, who remain *M.tb* uninfected despite the long-lasting high exposure to this pathogen [3, 4].

 The ability of *M.tb* bacilli to survive within host cells, especially monocyte/macrophages, accounts for their worldwide spread. Macrophages are the first cells to encounter *M.tb* and are designated to eliminate pathogens by phagocytosis and microbial killing. However, virulent TB bacteria are able to avoid host defense by colonization of macrophage nondegradative phagosomes [3, 5]. The interaction of macrophages with *M.tb* starts immediately on the contact between the pathogen and macrophage receptors. Key macrophage receptors that sense mycobacterial products include Toll-like receptors (TLRs), CD14 coreceptor, and C-type lectin receptors [6–8]. These receptors also sense endogenous signals resulting from tissue damage and necrosis [9]. The signaling program initiated by macrophage receptors results in the activation of transcription factors leading to the expression of inflammatory mediators, cytokines, and chemokines. The signals induced by CD14 and TLRs concomitantly activate feedback inhibitory mechanisms that restrain the magnitude of inflammatory signaling [10]. The dynamics of macrophage transition from an intracellular pathogen driven strong inflammatory signaling into a weak activated state may be important in the immune control of *M.tb* infection.

 The unique lipids composing the cell envelope of pathogenic mycobacteria such as lipoarabinomannan (LAM), lipomannan (LM), phosphatidylinositol mannoside (PIM), and trehalose 6,6′-dimycolate (TDM) interact with membrane CD14 (mCD14) and TLR2 on macrophages and activate signaling pathways inducing the innate immune response to infection [6, 7, 11, 12]. A soluble form of plasma CD14 (sCD14) is known to sensitize host cells to LPS; however, it also interacts with mycobacterial LAM causing upregulated endogenous CD14 gene expression [13]. Soluble sCD14 also activates the production of cytokines and adhesion molecules in CD14 negative cells, such as endothelial and epithelial cells, via LAM-sCD14 complexes. A role of CD14 in the phagocytosis of nonopsonized *M.tb* was suggested in the studies which demonstrated that both anti-CD14 mAb and soluble CD14 could significantly inhibit the uptake of *M.tb* bacteria by human microbial cells [14]. However, a major role in mycobacterial uptake has been attributed to the complement receptors and the mannose receptors (MRs) [15, 16]. The mycobacterial LAM has been shown to function as a ligand which is likely to mediate the uptake of *M.tb* via MRs on macrophages [7, 17, 18]. The serum mannose-binding lectin (MBL) is also an important part of innate immune response to mycobacteria. MBL opsonizes and facilitates phagocytosis of *M.tb* [19]. The serum concentration of MBL is significantly increased in patients with active TB [20]. During *in vivo* inflammatory response, there is a steady migration of monocytes from the circulation into inflammatory sites. The *β*2 integrin lymphocyte function associated antigen-1 (LFA-1) plays an important role in the trafficking of *M.tb* infected macrophages within the host [21]. Human macrophages infected with *M.tb* exhibit an increase in LFA-1 expression and the cell adhesive properties. Thus, modification of LFA-1 on *M.tb* infected cells may regulate homotyping cellular adhesion in granuloma formation and antigen presentation by influencing mutual interactions of *M.tb* activated macrophages with T cells [22, 23]. The indispensable requirement of LFA-1 for protective immunity during pulmonary tuberculosis has been demonstrated by Ghosh et al. [24].

 In our studies, we compared the expression of mCD14 and TLR2 signaling receptors, mannose receptor CD206 and LFA-1 molecule, on blood monocytes, and the serum concentration of sCD14, in patients with active pulmonary TB or nonmycobacterial lung diseases and in healthy individuals with latent *M.tb *infection detected by the IFN-*γ* releasing assay (IGRA) and the uninfected IGRA negative subjects. The relationship between the expression of monocyte and serum signaling receptors, skin tuberculin hypersensitivity, and IFN-*γ* response to the *M.tb* specific antigens in patients with active TB and nonmycobacterial infectious lung diseases has also been investigated.

## 2. Materials and Methods

### 2.1. Subjects

The study presented here has been approved by the Bioethics Committee of the Medical University in Lodz and written informed consent has been obtained from all study participants. The study cohort comprises 218 Poles, 43 patients with active pulmonary tuberculosis (TB patients), 41 household contacts (HTBCs) and 48 work contacts (WTBCs) of TB patients and 46 patients with nonmycobacterial lung diseases (NMLD), recruited at the Regional Specialized Hospital of Tuberculosis and Lung Diseases in Lodz. Forty-six healthy individuals with no history of tuberculosis and no known TB contact (Controls) were recruited from among acquaintances of the employees of the Institute for Microbiology and Immunology, University of Lodz. The patients underwent clinical examination, chest X-ray, sputum microscopy and culture on the Löwenstein-Jensen medium, and tuberculin skin testing. All TB patients had proven TB bacteriology (staining and/or culture) and typical chest X-ray findings. The NMLD patients were hospitalized for a common lower respiratory tract infection, pneumonia (36), pleurisy (5), or bronchitis (5). These patients had no history of TB and showed negative TB bacteriology. Taking into account the age range of the individuals included in the study (17–85) and the fact that since 1950 all newborns and school children in Poland have been obligatorily immunized with antituberculosis BCG (Bacille Calmette-Guérin) vaccine, there is a great probability that over 98% of the participants have been BCG vaccinated.

### 2.2. QuantiFERON-TB Gold in Tube (QFT-G) Assay

Peripheral blood samples were obtained from all participants. In the case of TB patients, they were collected at the time of diagnosis and the beginning of treatment. QFT-G test (Cellestis Limited, Carnegie, Australia) was performed according to the instructions of the manufacturer. Heparinised whole blood was incubated with a mixture of *M. tuberculosis* antigens (ESAT-6, CFP-10, TB 7.7 (p4)) or PHA mitogen or with no stimulant for 24 h at 37°C in an atmosphere enriched with 5% CO_2_. After the incubation, the tubes were centrifuged at 2500 RCF for 15 minutes at room temperature. The plasma was harvested above the gel plug and stored at −20°C until the immunoenzymatic determination of the IFN-*γ* level. Results were calculated and interpreted using QuantiFERON-TB Gold Analysis Software. An IGRA result was considered positive if the difference between the IFN-*γ* concentration in *M.tb* antigens-stimulated (TB Ag) and unstimulated (Nil) blood cultures was both ≥0.35 IU/mL and ≥25% of the Nil value. The quantitative IFN-*γ* results obtained by IGRA testing (TB Ag-Nil) were also calculated.

### 2.3. Flow-Cytometry Analysis of mCD14, TLR2, CD206, and LFA-1 Expression

**Figure 1:**
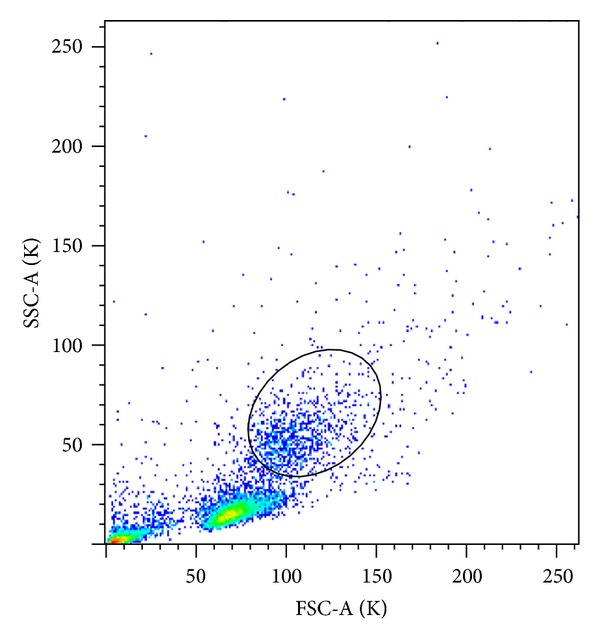
Gated monocytes on FSC-A/SSC-A plot.

Samples of heparinized blood from all participants were used for flow-cytometry analysis. The peripheral blood mononuclear leukocyte fractions (PBML) were separated by density gradient centrifugation (1200 RCF, 20 min, 20°C) on LSM 1077 (PAA Laboratories GmbH, Austria). The PBML at interface were harvested, washed twice with the RPMI-1640 medium (PAA Laboratories), and centrifuged at 250 RCF for 10 minutes at 20°C. Finally, PBML were suspended in PBS (Phosphate Buffered Saline) and the density of the cell suspension was adjusted to 5 × 10^6^ cells/mL. The cell (10^5^ cells) samples were incubated with the following monoclonal antibodies: mouse FITC-conjugated IgG2a anti-human CD14 (BD Biosciences Pharmingen, San Diego, USA), PE-conjugated IgG2a anti-human TLR2 (eBioscience, San Diego, USA), PE-conjugated IgG1 anti-human CD206 (BD Biosciences), and PE-conjugated IgG1 anti-human LFA-1 (BD Biosciences Pharmingen, San Diego, USA). Isotype control antibodies were used as control for nonspecific binding. After 30 min incubation with the appropriate antibodies, the cells were washed twice with PBS, centrifuged (520 RCF, 10 minutes, 4°C), and finally suspended in 200 *μ*L of PBS. The cells were analysed by flow cytometry using the FACScan (BD) and FlowJo software 7.2.2. (Tree Star Inc., USA). A total of 10,000 cells were analyzed. Forward and side scatter of PBML, the gated monocytes (Figure 1) were used for analysis of CD14 staining. The levels of TLR2, CD206, and LFA-1 expression were estimated in the cells gated for CD14(+) monocytes (Figure 1). The expression of the receptors on macrophages was calculated as mean fluorescence intensity (MFI) of samples treated with monoclonal antibodies diminished by MFI value of isotype matched negative controls. The percentage of the macrophages positive for investigated signal receptors was also analyzed.

### 2.4. Soluble CD14 (sCD14) in Serum

The concentration of sCD14 in the sera was measured by a specific ELISA assay with commercial Human sCD14 EIA Kit (R&D Systems, Minneapolis, MN, USA) according to the manufacturer's instructions. The optical density (OD) of each sample was determined using a multifunctional counter Victor 2 (Wallac Oy, Turku, Finland) set at 450 nm.

### 2.5. Tuberculin Skin Test

In TB and NMLD patients, the tuberculin skin test (TST) was applied using 2 units of Purified Protein Derivative (PPD RT 23, Statens Serum Institute, Copenhagen, Denmark) using the Mantoux method and indurations were measured using the palpation method 72 hours later. TST results were defined as negative (<10 mm) and positive (≥10 mm).

### 2.6. Statistical Analysis

The analysis of the results was performed using the Statistica 10.0 PL software program (Statsoft). Differences in the frequencies between studied groups were compared by Pearson Chi-squared (*χ* ^2^) test or two-tailed Fisher's exact tests. A probability value of 0.05 or less (*P* ≤ 0.05) was considered statistically significant. Comparisons between two groups were done by *U* Mann Whitney test and for more than two groups by Kruskal-Wallis Anova test. Correlations were assessed using the Spearman's correlation coefficient. Concordance between the TST and IGRA was analyzed using *κ* coefficient, where *κ* > 0.8 represents excellent agreement, *κ* values from 0.4–0.8 represent fair to good agreement, and *κ* < 0.4 represents poor agreement.

## 3. Results

### 3.1. The Infectious and Immune Status of Participants

To investigate a possible influence of TB lung disease and nonmycobacterial lung infection (NMLD) on the monocyte signaling receptors, a total of 224 volunteers were recruited into the study. Two groups of patients were under the care of the Regional Specialized Hospital of Tuberculosis, Lung Diseases and Rehabilitation. They were characterized as follows: patients with active TB (43) microbiologically confirmed by a sputum culture and typical chest X-ray findings, patients with NMLD (46), with no history of TB, who had negative TB bacteriology and a chest X-ray interpreted as nontuberculous pneumonia (36), pleurisy (5), or bronchitis (5). Over 98% of patients had been vaccinated with BCG at birth and school age. The TST performed for the two groups of patients considered positive when induration was ≥10 mm. Nearly 56% TB patients classified as TST positive versus 28% in the NMLD group (*P* = 0.008) (Table 1).

The second focus of the study was to analyze and compare the expression of monocyte signaling receptors in individuals with active or latent tuberculosis and without TB infection. The T-cell-based IFN-*γ* release assay (IGRA) was used to detect latent *M. tuberculosis *infection in healthy volunteers with no TB history: 41 household TB contacts, 48 healthcare workers in the tuberculosis sector (work TB contacts), and 46 community controls (Controls) who did not have a known contact with TB. The IGRA was also performed in NMLD and TB patients. A similar prevalence of positive IGRA was found in healthy Controls (13%) and NMLD patients (14%) (Table 1). The highest prevalence of latent TB infection (44%) was observed in work TB contacts, mainly females working in the TB sector for years. A lower prevalence rate 27% was showed in household TB contacts who were recruited for the study shortly after confirming active TB in their relatives. Thus, household TB contacts could have been suspected of a recent exposition to *M.tb* bacteria. The T-cell-based IFN-*γ* release assay was positive in 65% patients with active TB and in 13% of NMLD patients. Thus, the sensitivity of the QuantiFERON-TB Gold In Tube assay for TB diagnosis in patients with lung diseases was 65%, while the specificity was 87%. IGRA showed higher sensitivity (65% versus 55%) and specificity (87% versus 72%) compared to TST (positive predictive value 82% versus 65% and negative predictive value 73% versus 63%).

The rate of positive results for both TST and IGRA was higher among TB (46%) than in NMLD (9%) patients (*P* < 0.001) (Table 2). In contrast, 67% NMLD patients versus 26% TB patients had negative both TST and IGRA results (*P* < 0.001). However, no significant difference in the rate of discordant TST and IGRA results was observed between the two groups of patients (*P* > 0.05). The overall agreement between the TST and IGRA in all patients was moderate (*κ* = 0.46). The TST and IGRA concordance rate was also moderate in TB patients (*κ* = 0.41), but it was poor in NMLD patients (*κ* = 0.27) (Table 3).

**Figure 2:**
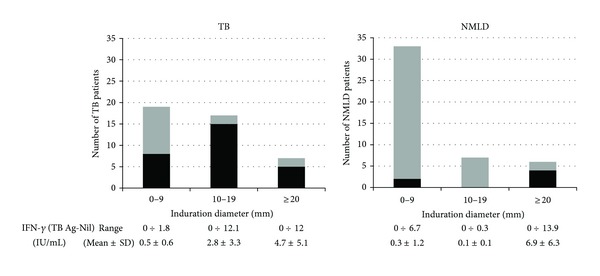
Distribution of induration diameters in TB and NMLD patients. Bars represent the number of patients with different TST size, showing either a IGRA positive (black) or a IGRA negative (gray).

Figure 2 shows the distribution of IGRA positive results according to the induration size of TST in TB and NMLD patients. The majority of IGRA positive TB patients (71.4%) showed the induration size of equal or greater than 10 mm. Four out of six IGRA positive NMLD patients showed extensive skin induration larger than 20 mm. To perform more precise analysis of the relationship between the PPD driven TST and *M.tb* stimulated IFN-*γ* release, we calculated the cytokine concentration in whole blood cultures with ESAT-6, CFP-10, and TB 7.7. The results obtained by the Nil control were subtracted from the *M.tb* antigen-stimulated samples (TB Ag-Nil IU/mL) (Figure 2). Analysis revealed a correlation between the induration diameter of skin reaction to PPD and the IFN-*γ* release in response to specific *M.tb* antigens, in the TB group (*P* < 0.001, *r* = 0.51) but not in NMLD patients (*P* > 0.05, *r* = 0.22).

**Figure 3:**
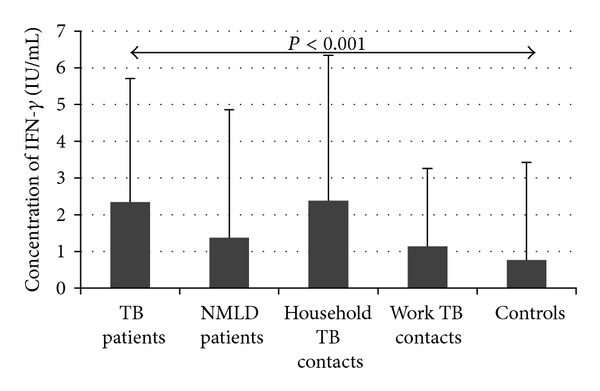
The mean quantity of IFN-*γ* in all responders to specific *M.tb* antigens used in IGRA.

The quantitative IFN-*γ* results obtained by IGRA testing (TB Ag-Nil) were also analyzed. Comparing the IFN-*γ* responses in all groups, we found that the mean of IFN-*γ* response values were significantly higher in patients with active TB and healthy work contacts exposed to *M.tb* for a long time as compared to healthy Controls with no known contacts to infectious TB (Table 4). In contrast, there were no differences in the mean values of IFN-*γ* production in healthy Controls and household recent contacts of patients with active TB and in patients with nonmycobacterial lung disease. It should be emphasized that the intergroup differences in the mean quantities of IFN-*γ* responses were, at least partly, caused by different frequency of responses to specific *M.tb* antigens used in IGRA (Table 4). When analyzing all responders, a significant increase in the quantity of released IFN-*γ* was noticed only in patients with active TB (Figure 3).

### 3.2. The Flow Cytometry Analysis for Membrane-Bound CD14 (mCD14) on Freshly Isolated Monocytes

**Figure 4:**
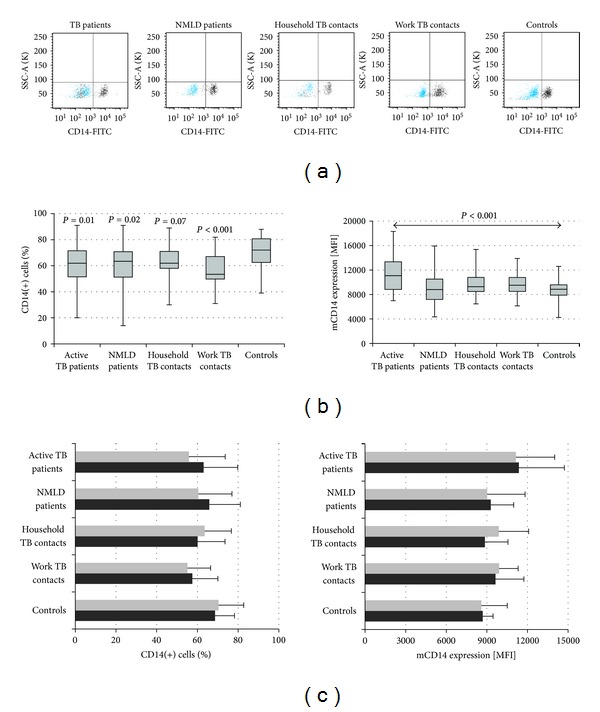
CD14 surface expression of monocytes. (a) Representative flow plots; (b) median and interquartile range in percentage (left) and mean fluorescence intensity (MFI) (right) in each group; (c) MFI values in IGRA negative (grey) and positive (black) participants in each group.

Assuming that the percentage of CD14(+) monocytes recorded in healthy Controls represents normal value, a significant increase in the proportion of this cell fraction was found in TB and NMLD patients and healthy work TB contacts (*P* < 0.02). In household contacts, only a trend to increase the proportion of CD14 positive monocytes was noticed (*P* = 0.07) (Figure 4(b), left). While analyzing the MFI values of membrane bound CD14 receptors on monocytes, a significant overexpression of mCD14 was found only in patients with active TB as compared to Controls (*P* < 0.001) (Figure 4(b), right).

**Figure 5:**
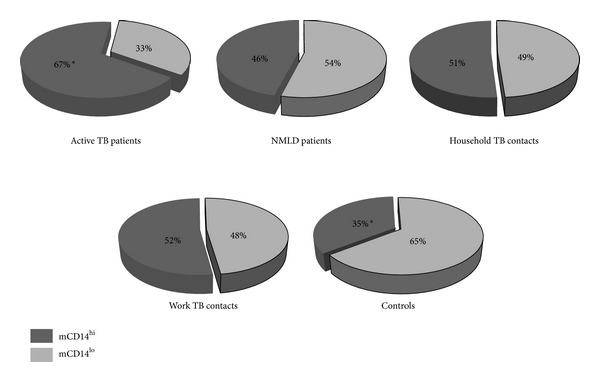
The percentage of monocytes designated CD14 high (CD14^hi^) and CD14 low (CD14^lo^) in the study groups. *significantly different TB patients from Controls (*P* = 0.002).

To extend the analysis of the alteration in the CD14 expression on monocytes in the study groups, we used median MFI value as an arbitrary base to calculate the proportion of monocytes with CD14 MFI above median, which were designated CD14 high (CD14^hi^) and monocytes with CD14 MFI below median designated CD14 low (CD14^lo^).  Figure 5 records the proportion of two monocyte subsets in the study groups. Patients with active TB presented significantly higher frequency of CD14^hi^ monocytes (67%) as compared to healthy Controls (35%) (*P* = 0.002). The proportion of CD14^hi^ monocytes in healthy TB contacts and NMLD patients was slightly higher than in the Controls (51%, 52% and 46 resp.), however, the differences were not significant (*P* > 0.05).

The distribution of CD14^hi^ and CD14^lo^ monocytes was similar in IGRA negative and positive patients with active TB and their contacts. A small number of IGRA positive tests in healthy Controls and NMLD patients did not allow for proper calculation of the association between these two immunological parameters.

### 3.3. Soluble CD14 (sCD14) in Sera

**Figure 6:**
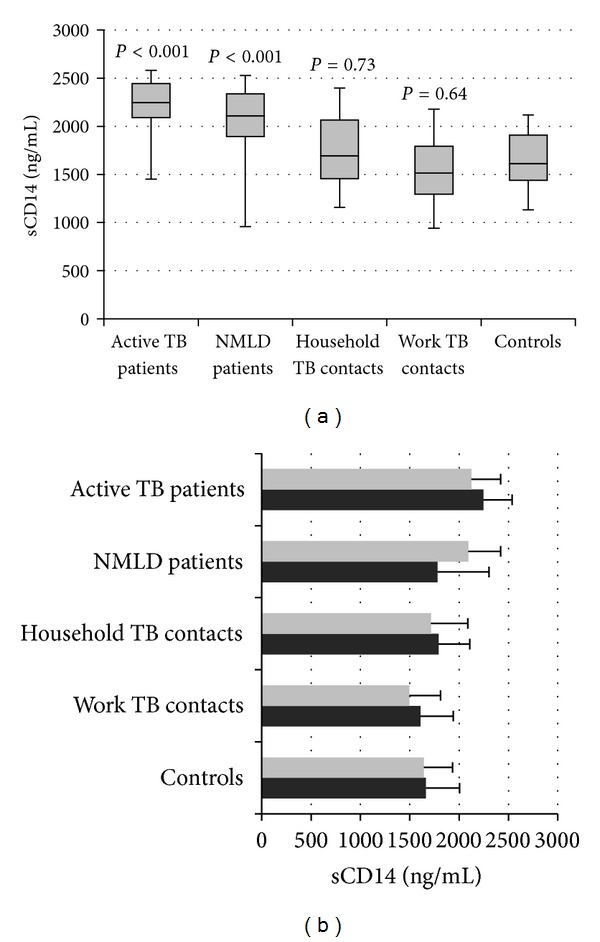
The serum concentration of sCD14 in the study groups (a) characterized by positive or negative IGRA (b).

In parallel with the flow cytometry analysis for CD14 expression on monocytes, we determined the concentration of soluble sCD14 in the sera examined by ELISA. The levels of serum sCD14 in patients with active TB and nonmycobacterial lung infections were significantly elevated in comparison to Controls (*P* < 0.001) (Figure 6(a)). In contrast, similar levels of sCD14 were detected in the sera from healthy household and work TB contacts and Controls with no known TB contact (*P* > 0.05). The results also revealed no differences in the sCD14 concentration between IGRA positive and IGRA negative individuals in the study groups (Figure 6(b)). Finally, we found no correlation between the expression of membrane bound mCD14 and serum concentration of soluble sCD14 molecules in the study groups: TB patients (*r* = 0.26, *P* = 0.09), TB contacts (*r* = 0.22, *P* = 0.11), Controls with no known TB contact (*r* = 0.15, *P* = 0.30), and NMLD patients (*r* = 0.21, *P* = 0.18).

### 3.4. The Flow Cytometry Analysis for TLR2 Receptors on Freshly Isolated Monocytes

**Figure 7:**
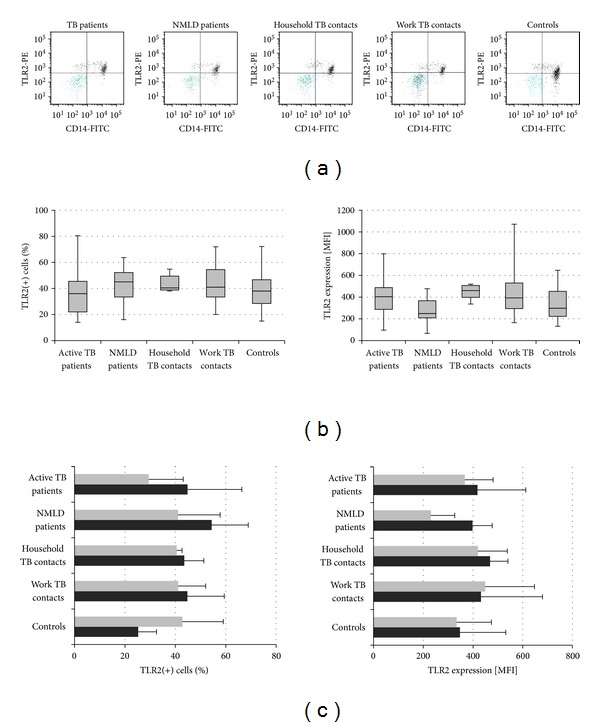
TLR2 surface expression of CD14(+) monocytes. (a) Representative flow plots; (b) median and interquartile range in percentage (left) and mean fluorescence intensity (MFI) (right) in each group; (c) MFI values in IGRA negative (grey) and positive (black) participants in each group.

The significant increase in mCD14 in patients with active TB prompted us to investigate the expression of TLR2 receptors forming heterodimers with CD14 in monocytes, which increases the diversity of molecules recognized by the receptors [25]. As an accessory molecule CD14 loads mycobacterial cell wall constituents, lipomannan or lipoarabinomannan, onto TLR2/TLR1 heterodimers, which is an initial step in a cascade of events leading to cell activation [26, 27]. Our results showed similar frequencies of CD14(+) monocytes carrying TLR2 receptors in all study groups (Figure 7(b), left), in both IGRA negative and positive volunteers (Figure 7(c), left). Also, there were no intergroup differences in TLR2 MFI values in CD14(+) monocytes (Figure 7(b), right) from TB and NMLD patients and healthy participants with or without contacts to infectious TB, both IGRA negative and positive participants (Figure 7(c), right).

### 3.5. The Flow Cytometry Analysis for CD206 Receptors on Freshly Isolated Monocytes

**Figure 8:**
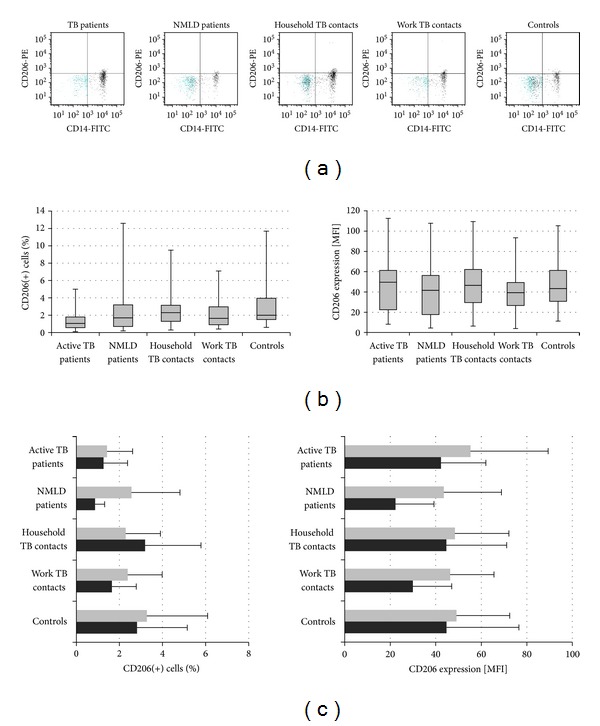
CD206 surface expression of CD14 (+) monocytes. (a) Representative flow plots; (b) median and interquartile range in percentage (left) and mean fluorescence intensity (MFI) (right) in each group; (c) MFI values in IGRA negative (grey) and positive (black) participants in each group.

The entry of *M.tb* bacilli into host mononuclear cells and their survival and replication within these cells is the first step leading to TB infection [28]. The mCD14 is ligand delivery and an enhancer of TLR2 signaling. In 1995, Peterson et al. [29] suggested that CD14 receptors could facilitate entry of nonopsonized TB bacilli into macrophages within the brain. However, a later report by Shams et al. concluded that CD14 did not mediate the entry of *M.tb* in human alveolar macrophages [30]. Instead, it has been demonstrated that *M.tb* enters human macrophages through complement receptors, mannose receptors, scavenger receptors, and the surfactant protein A receptor [31, 32]. In this study, we compared the expression of the CD206 mannose receptor on monocytes from volunteers with active or latent TB, nonmycobacterial lung infections, and matched Controls. We could detect no significant intergroup differences in the proportion of CD14(+) monocytes with CD206 expression (Figure 8(b), left), in both IGRA negative and positive participants (Figure 8(c), left). Also, the CD206 MFI values observed in TB and NMLD patients and healthy volunteers with or without contacts to infectious TB were similar (Figure 8(b), right), in both IGRA negative and positive (Figure 8(c), right) participants.

### 3.6. The FLow Cytometry Analysis for LFA-1 Integrin on Freshly Isolated Monocytes

**Figure 9:**
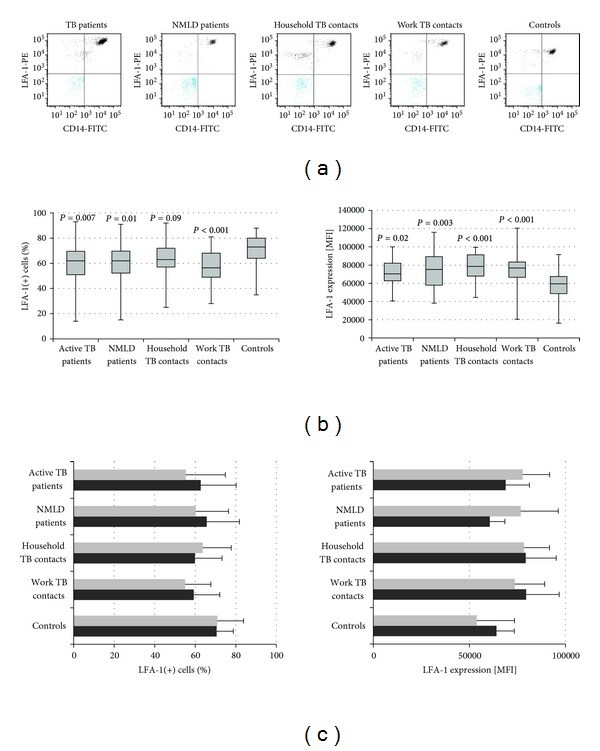
LFA-1 surface expression of CD14(+) monocytes. (a) Representative flow plots; (b) median and interquartile range in percentage (left) and mean fluorescence intensity (MFI) (right) in each group; (c) MFI values in IGRA negative (grey) and positive (black) participants in each group.

Following the entry of *M.tb* bacilli into macrophages, the innate and particularly adapted immune responses against tuberculosis depend on the trafficking of *M.tb* infected macrophages within the host. The *β*2 integrin lymphocyte function associated antigen-1 (LFA-1) plays an important role in this process [21]. The analysis of flow cytometry data for LFA-1 integrin revealed that healthy Controls with no known TB contact possessed significantly higher proportion of LFA-1 positive monocytes as compared to TB and NMLD patients and work contacts of TB patients, although in household contacts only a trend to increase was noticed (Figure 9(b), left). In contrast, LFA-1 MFI value was significantly lower in Controls than in the other study groups (Figure 9(b), right). These data could suggest that monocyte LFA-1 expression is a sensitive biomarker of infections from several lung pathogens. Further analysis revealed a positive correlation between LFA-1 and mCD14 MFI values in all participants (*P* < 0.001, *r* = 0.28) and in each study group (0.00002 < *P* < 0.03,  0.31 < *r* < 0.57) excluding patients with nonmycobacterial lung disease.

**Figure 10:**
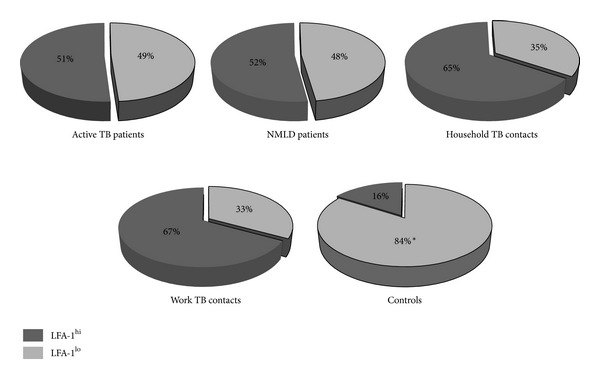
The percentage of monocytes designated LFA-1 high (LFA-1^hi^) and LFA-1 low (LFA-1^lo^) in the study groups. *significantly different Controls from TB and NMLD patients and household and work cotacts to TB infection (*P* < 0.001).

To extend the analysis of the alteration in the LFA-1 expression on monocytes in the study groups, we used median MFI value as an arbitrary base to calculate the proportion of CD14(+) monocytes with LFA-1 MFI above median which were designated LFA-1 high (LFA-1^hi^) and monocytes with LFA-1 MFI below median designated LFA-1 low (LFA-1^lo^).  Figure 10 records the proportion of two monocyte subsets in the study groups. Patients with active TB or nonmycobacterial lung disease as well as healthy household and work contacts presented significantly higher frequency of LFA-1^hi^ monocytes as compared to healthy Controls (*P* < 0.001).

The distribution of LFA-1^hi^ and LFA-1^lo^ monocytes in IGRA negative and positive patients with active TB and their contacts did not differ significantly. A small number of IGRA positive tests in healthy Controls and NMLD patients did not allow for proper calculation of the association between these two immunological parameters.

### 3.7. The Relationship between IFN-*γ* Production in IGRA, the Expression of Signal Transduction Receptors on Monocytes, and Delayed Type Hypersensitivity (DTH) to PPD

The quantity of IFN-*γ* produced in response to *M.tb* specific antigens in IGRA was calculated as TB Ag-Nil. Then, we have analyzed the relationship between the IFN-*γ* response levels in whole blood cultures stimulated with the combination of ESAT-6 + CFP-10 + TB 7.7 antigens, and the expression of signal transduction receptors on monocytes measured as MFI values. The analysis revealed no association between IFN-*γ* responses to *M.tb* specific antigens and MFI values of mCD14, TLR2, CD206, and LFA-1 receptors either in patients with active TB, healthy volunteers with latent TB suggested by IGRA positive results, or healthy IGRA negative participants.

## 4. Discussion

Many studies have demonstrated a crucial role of macrophage pattern recognition receptors in intracellular survival and replication of virulent mycobacteria, delay in the onset of T cell responses, and pathogenesis generated by these bacteria [7, 30, 33]. Our strategy was to determine the expression of signal transduction receptors in patients with active lung TB and individuals with or without latent TB to explain the background of the differences between clinical and subclinical forms of *M.tb* infection. We detected a significant increase in the expression of CD14 receptors in patients with active TB as compared to healthy Controls. This increase was documented as the enhancement in monocyte CD14 MFI values, considerable rise in frequency of monocytes characterized by elevated mCD14 expression (CD14^hi^) and significant increment in the concentration of soluble sCD14 molecules in the serum. No increase in mCD14 MFI values was seen in healthy household and work contacts to infectious TB. The marked enhancement in CD14 expression in patients with active TB could have resulted from the activity of virulent *M.tb* contributing to TB pathogenesis. It should be emphasized that in TB patients the increased CD14 expression on monocytes was accompanied by the increase in the level of *β*2 integrin lymphocyte function associated antigen-1 (LFA-1). The elevated coexpression of these two molecules suggests the importance of CD14-LFA-1 signaling in TB infections when the immune control is broken by *M.tb* bacilli, which leads to active TB. A possible involvement of the CD14-LFA-1 signaling in the transition of latent *M.tb* infection into active disease cannot be excluded. The immune control of mycobacterial infection depends positively on the formation and maintenance of granuloma [34]. Granuloma are the sites where virulent mycobacteria are allowed to persist in a state of low metabolic activity [35]. After decades of dormancy, *M.tb* bacilli can reactivate and cause necrosis of granuloma, which facilitates bacterial repopulation and dissemination in spite of existing adapted immunity. The CD14-LFA-1 signaling is likely to contribute to *M.tb* driven shift of protective towards destructive function of granuloma. The mCD14, a 55-kD glycoprotein serves as a pattern recognition receptor for mycobacterial components such as, LAM, LM, PIM, TDM, and lipoproteins [6–8, 11]. The interactions of mycobacterial constituents with both membrane bound mCD14 and soluble sCD14 lead to the activation of transcription factors, production of proinflammatory cytokines/chemokines, and upregulation of cell adhesion molecules, which are a molecular basis for development of adapted immunity and granuloma [36]. However, an excessive inflammatory reaction to *M.tb* infection may induce the aggressive granulomatous response which could be conducive to pathological reactions and symptoms of active TB. This suggestion has been confirmed by our study where patients with active TB showed greater production of IFN-*γ* in response to specific *M.tb* antigens compared to healthy Controls (Figure 3). Moreover, in patients with active TB, we noticed a positive correlation between IFN-*γ* responsiveness to specific *M.tb* antigens, ESAT-6, CFP-10 and TB 7.7, and intensity of skin reactivity to PPD (Figure 2). The tuberculin skin test measures delayed type hypersensitivity to mycobacterial antigens and IFN-*γ* is the main cytokine which regulates positively the development of DTH reaction [37, 38]. IFN-*γ* itself may also increase the expression of LFA-1 on murine macrophages [39]. The simultaneous increase in the expression of mCD14 and LFA-1 on monocytes from patients with active TB could have enhanced the inflammatory response to mycobacterial components. The LFA-1 integrin plays an important role in the trafficking of *M.tb* infected macrophages within the host and in the initiation of the immune synapse between an *M.tb* infected macrophage and a T lymphocyte [21, 34, 35]. A requirement of LFA-1 for protective immunity during pulmonary *M.tb* infection has been documented in animal models by Turner et al. [40] and Ghosh et al. [24]. As an adhesion molecule LFA-1 contributes to the cellular migration and cell-cell interaction necessary for granuloma formation [41]. On the other hand, enhanced expression of this receptor on macrophages may lead to disaggregation of the granuloma and reactivation of latent *M.tb* infection. 

In this study, the enhancement in the monocyte mCD14 level was observed only in patients with active TB (Figure 4). In contrast, the increase in LFA-1 MFI values above the values recorded in healthy Controls with no known exposition to cases of active TB was observed on monocytes from patients with active TB and their healthy contacts but also on monocytes from patients with nonmycobacterial lung disease. Thus, the increase in LFA-1 expression could be considered as a common marker of any infection. DesJardin et al. [21] observed increased expression of LFA-1 and its counter receptor ICAM-1 on *M.tb*—infected human monocyte—derived macrophages, which confirms our data. This increase resulted in an enhanced cell clustering, a decrease in surface levels of the phagocytic receptors CR3, CR4 and Fc*γ*RII, and an increase in major histocompatibility complex Class II molecules. The decrease in surface levels of complement receptors CR3 and CR4 was correlated with a diminished phagocytic capacity of macrophages. 

In comparison to Controls with no known contacts to infectious TB, a significant decrease in the proportion of CD14(+) monocytes (Figure 4(b), left) and CD14(+)LFA-1(+) (Figure 9(b), left) monocytes was found in patients with active TB or nonmycobacterial lung diseases and in work contacts exposed to *M.tb* for a long time. Thus, the decrease in the frequency of CD14(+) and CD14(+)LFA-1(+) monocytes could reflect a lasting lung infection from any pathogen not exclusively mycobacteria. The decreased frequency of monocytes with mCD14 and LFA-1 coexpression in PBML from TB and NMLD patients could have been caused by intensive recruitment of such cells into lungs. Indeed, a significant difference between profile of *M.tb* driven immune response in lung and periphery was observed by Chiacchio et al. [42]. A possible recruitment of mCD14 and LFA-1 monocytes into lungs of the healthcare workers in the tuberculosis sector who carried replicating TB bacilli in the absence of clinical symptoms should be considered. The increased mean IFN-*γ* value of responses to *M.tb* specific antigens, which was observed in this group, confirms our suggestion. In contrast to work contacts exposed to *M.tb* for a long time, no significant decrease in the percentage of CD14(+) and CD(14+)LFA-1(+) monocytes and the LFA-1 MFI values was recorded in the group of healthy household recent contacts of patients with active TB. It is likely that the period between the exposition to *M.tb* and volunteers examination was too short to determine any differences in investigated parameters in comparison to Controls. 

We have previously showed that a significant increase in serum sCD14 characterizes patients with active TB [20]. This observation was confirmed by other authors [43, 44], which allowed recognizing increased sCD14 as a potential biomarker of TB disease. Consistent with our previous investigations, in this study, a significant increase in sCD14 was found for TB patients. However, additional investigations conducted by us revealed a considerable sCD14 increase in patients with nonmycobacterial lung diseases. This was not surprising because sCD14 is an acute-phase protein, acting as a negative regulator of human T cell activation and function [45]. Elevated serum sCD14 levels were found in many noninfectious (Crohn's disease, SLE) or infectious (HIV, chlamydiosis) diseases [46–49]. It is reasonable to think that CD14-159C/T polymorphism might have accounted for the differences in the CD14 expression resulting from variable macrophage responses to host endogenous and bacterial stimuli. On the other hand, it has been suggested that in chronic inflammatory diseases enhanced shedding of mCD14 from the bacteria-activated monocyte membrane is associated with a higher concentration of sCD14 in the serum [50]. In patients with acute nonmycobacterial lung diseases the sCD14 increase was not accompanied by an enhancement in the expression of mCD14 on monocytes. It is quite likely that endothelial cells could have been a source of sCD14 increase in NMLD patients developing an inflammatory response to bacterial infections. It has been suggested that large amounts of sCD14 in circulation could come from CD14(+) hepatocytes and endothelial cells [51, 52].

Multiple components of mycobacteria such as peptidoglycan, lipoteichoic acid, LAM, PIM, and TDM initiate intracellular signaling in CD14- and TLR2-dependent manner [6, 7, 11, 12, 53, 54]. The mannose-capped LAM of virulent *M.tb* complex bacilli has been established as an agonist for TLR2 [26]. TLR2 activation leads to the killing of intracellular *M.tb* in both mouse and human macrophages [55]. In humans, TLR2 polymorphisms that decrease TLR2 expression were found to predispose people to tuberculosis [56]. However, we could see no difference in the TLR2 expression on monocytes from TB or NMLD patients and healthy volunteers with or without contacts to infectious TB. It can be partly explained by ubiquitous to all bacteria TLR2 ligands, diacyl and triacylglycerol moieties, proteins, and polisacharides [25]. It is also known that mycobacterial cell wall components may activate cells through the combined actions of TLR2 and TLR1 or TLR4 [26, 53, 54, 57, 58]. The TLR4 signaling was required to mount a protective response during chronic *M.tb* infection in mice [57]. Thus, including TLR1 and TLR4 into the study could have increased the knowledge of the role of TLRs in active and latent TB. 

The macrophage mannose receptor CD206 plays a major role in the uptake of *M.tb* bacilli by macrophages [15, 16]. Ligation of the macrophage CD206 by mycobacterial mannose-capped LAM or PIM is associated with an activation of anti-inflammatory cytokines and limitation of oxidative response, which may promote *M.tb* infection. These CD206 attributes prompted us to include this receptor in the study. However, we observed no intergroup differences in its expression. The lack of intergroup differences in CD206 expression and significant alterations in the expression of mCD14 and LFA-1, which we observed in the participants differing in the aspects of *M.tb *infection and health, confirm the opinion that mycobacterial components use divers Toll-like receptors pathways to induce intracellular signaling cascades [53, 54]. 

In mycobacterial infections important pro-inflammatory activity is attributed to IFN-*γ*. On the whole, greater mean IFN-*γ* responses to *M.tb* specific antigens are shown in active than latent TB [59, 60]. In our study, patients with active TB showed also the greatest production of IFN-*γ* when examined by IGRA. However, the cytokine production in healthy work contacts exposed to *M.tb* for a long time was also higher than in household recent contacts to infectious TB. It has been suggested that subjects with high levels of IFN-*γ* production in response to ESAT-6 and/or CFP-10 antigens in IGRA have a higher possibility of developing active TB than IGRA positive subjects with lower levels of IFN-*γ* [61]. The enhanced IFN-*γ* responses in work contacts to infectious TB may also suggest that a long exposition to TB bacilli is essential for developing specific Th1 response. The group of patients with active TB is distinguished from others by the simultaneous increase in the monocyte mCD14 and LFA-1 MFI values. However, there was no correlation between the alterations in the expression of these receptors and IFN-*γ* responses in IGRA. The lack of correlation between these parameters may be explained by a profound difference in the repertoire of *M.tb* antigens “pulsing” immune cells in patients with active or latent TB and a combination of three *M.tb* specific antigens used in IGRA. Moreover, although IFN-*γ* mediates mycobacteria driven immune reactions, there are other cytokines such as IL-12, IL-18, IL-23, TNF-*α*, which may be involved in modulating the expression of signal transduction receptors on host macrophages.

## 5. Conclusions

The development of TB clinical symptoms usually results from endogenous reactivation taking place long after the exposure to *Mycobacteria tuberculosis*. The key question is which host defense mechanisms fail allowing the pathogen to multiply rapidly and cause clinical symptoms. A significant increase in the CD14-LFA-1 signaling in patients with active TB partly answers the question. The observed alterations in the expression of the membrane-bound mCD14 receptor and in the frequency of monocytes with a high expression of this receptor may be considered as a biomarker of active TB disease. A continuation of the study, particularly in the group of contacts of patients with active TB, might allow the better understanding of the relationship between the alterations in the expression of signal transduction receptors and risk of developing active TB in *M. tuberculosis *infected subjects.

## Figures and Tables

**Table 1:** Characteristics of the groups in the study.

Clinical characteristics	Participants				
	Patients		Healthy individuals		
	TB patients	NMLD patients	Household TB contacts	Work TB contacts	Controls
Total number of subjects	43	46	41	48	46
Mean age in years (range)	48 (21–81)	52 (19–85)	38 (17–70)	44 (29–60)	47 (18–77)
Sex (no. (%) female)	19 (44%)	31 (67%)	21 (51%)	45 (93%)	30 (65%)
Ethnicity	Caucasians	Caucasians	Caucasians	Caucasians	Caucasians
BCG vaccination (%)	100%	100%	100%	100%	100%
Tobacco smoking (no. (%))	25 (58%)	11 (23%)	2 (13%)	3 (12%)	16 (34%)
Alcohol abuse (no. (%))	11 (25%)	3 (6%)	0	0	0
Smear^+^ and culture^+^ (%)	100%	0%	nd	nd	nd
IGRA result (no. (%))					
Negative	15 (35%)	40 (86%)	30 (73%)	27 (56%)	40 (87%)
Positive	28 (65%)	6 (14%)	11 (27%)	21 (44%)	6 (13%)
TST result (no. (%))			nd	nd	nd
Negative	19 (44%)	33 (72%)			
Positive	24 (56%)	13 (28%)			

nd: not done.

**Table 2:** Classification of TB and NMLD patients according to tuberculin skin test and IGRA results.

Test findings	TB patients *n* = 43	NMLD patients *n* = 46	*P* value*
TST+	24 (56%)	13 (28%)	*P* = 0.008
IGRA+	28 (65%)	6 (14%)	*P* < 0.001

TST+IGRA+	20 (46%)	4 (9%)	*P* < 0.001
TST+IGRA−	4 (9%)	9 (20%)	*P* > 0.05
TST−IGRA+	8 (14%)	2 (4%)	*P* > 0.05
TST−IGRA−	11 (26%)	31 (67%)	*P* < 0.001

*Comparison between results of TST and IGRA.

**Table 3:** Agreement between the TST and IGRA in TB and NMLD patients.

Groups	Concordance rate %	*κ*-value*	95% CI
All patients	74%	0.46	0.24, 0.67
TB patients	72%	0.41	0.10, 0.72
NMLD patients	76%	0.27	−0.14, 0.68

*κ coefficient.

**Table 4:** The production of IFN-*γ* in response to *M.tb* specific antigens.

Groups	IFN-*γ* (IU/mL)			
	Responders/group (%)	Range	Mean ± SD*	*P* value**
TB	38/43 (88%)	0–12.1	2.07 ± 3.25	<0.001
NMLD	32/46 (69%)	0–13.9	0.95 ± 2.96	0.4
Household TB contacts	24/41 (58%)	0–12.0	1.39 ± 3.23	0.29
Work TB contacts	41/48 (85%)	0–12.7	0.99 ± 2.01	<0.001
Controls	20/46 (43%)	0–12.0	0.33 ± 1.76	

*Mean ± SD values of cytokine in whole bood cultures stimulated with *M.tb* antigens (TB Ag-Nil);  **significance value between the responses in controls and each group of participants.
